# Supplementary material for: Are carbon emissions trading and green financial instruments synergistic? -Comprehensive quantitative research based on content analysis
Source: PLoS One. 2024 Mar 7;19(3):e0298601. doi: 10.1371/journal.pone.0298601 (PMC10919601; doi:10.1371/journal.pone.0298601)
Supplement: S1 Data — (ZIP) [file pone.0298601.s001.zip › Data/地级市Z_F工作报告.zip!/╒π╜¡/╬┬╓▌╩╨/└┤╫╘╣1⁄2╓┌║┼í╛┬φ┐╦╩2╛▌═°í┐_2015.docx]

各位代表：

　　现在，我代表温州市人民政府，向大会作工作报告，请予审议，并请市政协委员和其他列席人员提出意见。

一、2014年主要工作

　　过去一年，面对复杂的经济金融局面，我们在省委、省政府和市委的坚强领导下，负重前行、奋力赶超，深入实施“十大举措”，扎实推进“两美”建设，着力抓改革、抗风险、稳增长、促转型、强统筹、治环境、惠民生，较好地完成了市十二届人大四次会议确定的目标任务。实现地区生产总值4303亿元，同比增长7.2%；一般公共预算收入353亿元，增长8.8%；城镇常住居民人均可支配收入40510元，农村常住居民人均可支配收入19394元，分别增长8.7%、10.5%；居民消费价格涨幅控制在1.8%；城镇登记失业率1.91%；节能减排超额完成省下达任务。

　　（一）改革开放多点突破。“四张清单一张网”改革率先完成。市级部门行政权力从12723项减少到4168项，精减幅度居全省前列。市县政府责任清单公布实施。开展负面清单外企业投资项目不再审批试点。制定实施市级财政专项资金管理清单，出台非税收入项目目录。率先开通五级政务服务网。首创政府职能向社会组织转移“第五张清单”，首批167项政府职能、170项购买服务事项公布实施。全面深化改革有序推进。《温州市民间融资管理条例》全面实施，温州民商银行正式创立，“蓝海股份”一期、“幸福股份”三期成功发行；基本完成村级集体经济组织股改、土地承包权证到户，土地经营权流转率达63.7%；成为全国社会组织建设创新示范区，列入全国养老服务业综合改革试点；深化社会力量办学、办医改革，民办社会事业发展良好。新争取国家级改革试点22项、省级改革试点23项。着力抓好要素市场化配置改革，乐清、瑞安列入省级扩面试点；全面推进市场监管体制和商事制度改革，新登记企业数增长20.7%。出台深化国有企业改革意见，探索混合所有制改革，试行政府和社会资本合作模式。对外开放取得重大进展。温州港口岸实现扩大开放，保税物流中心（B型）、乐清湾港区水陆域开放、大陆居民赴台“个人游”获批，苍南霞关港成为更开放对台小额贸易点。成功承办浙台合作周活动，获批海峡两岸少数民族交流与合作基地；积极谋划温州韩国产业园，成功签约医乐园、韩国时尚新天地等项目，对台湾地区和对韩国合作开启新篇章。

　　（二）经济运行稳中向好。28项主要经济增长指标中，在全省位次前移的有17项，居全省末位的只剩4项，较2013年减少了4项。坚持投资、消费、出口一起上。大力实施“赶超发展三年超万亿投资计划”，限上固定资产投资3053亿元，增长16.6%，总量保持全省第三。实施温商回归“领头雁工程”，向10个省市派驻招商专员，发动异地温州商会组团回乡投资，实际到位内资969亿元。积极培育信息（网络）消费、时尚消费、月光经济等消费热点，社会消费品零售总额2410亿元，增长12.8%，为近三年来最高。出台促进开放型经济发展政策，外贸进出口208亿美元，增长0.9%，较2013年略有回升。坚持农业、工业、服务业一起抓。农业“两区”建设提速提效，粮食生产实现面积、总产、单产“三增长”，一产增加值增速升至全省第三。掀起工业强市建设高潮，制定实施助企强工“十条新政”，限上工业投资增长24%，居全省第一；规上工业增加值增长6.2%，逐步接近全省平均水平。召开现代物流业发展大会，一批大型商贸设施投用；泰顺廊氡、文成天湖省级旅游度假区获批，洞头国家级海洋公园规划通过评审，全市旅游总收入增长17.8%。坚持企业风险、金融风险、房地产风险一起治。推出“双十条”升级版，市县两级处置办实体化运作，帮扶315家重点困难企业化解资金链风险，政府应急转贷金循环使用达173亿元，银行不良贷款余额、不良率实现“双降”。落实税费减免缓政策，减轻企业负担62亿元。调整完善房地产调控政策，房地产市场趋于理性。

　　（三）转型升级成效显现。深入实施“五一○产业培育提升工程”。制定出台十大新兴产业发展规划、行动计划和配套政策，加快五大传统支柱产业改造提升，电气产业率先成为千亿级现代产业集群。确立时尚产业作为温州最具标志性产业的战略地位。创成全国质量强市示范城市。浙南沿海先进装备产业集聚区发展规划获批，南车车辆修造基地等一批超20亿元产业大项目落户瓯江口产业集聚区和温州经济技术开发区。开工建设小微企业园61个、建筑面积812万平方米。大力推进科技创新。完善国家级高新区管理体制。新增高新技术企业113家、科技型中小企业630家，入选省级企业研究院12家、研发（技术）中心30家，新产品产值增长39.9%。财政科技经费支出增长13.1%，发明专利授权量增长39.7%。申报“国千”60人，入选“省千”21人，均创历史新高。预计研究与试验发展经费支出占生产总值比重达1.62%。科技进步综合评价升至全省第四。全市首支国家级产业创投基金落户瓯海，瑞安获批国家知识产权试点县。积极推进“四换三名”。获批创建国家电子商务示范城市，网络零售额增长69%；工业技改投资增长56.1%，居全省第一；启动城镇低效用地再开发2.2万亩，盘活存量建设用地1.3万亩；淘汰行业性落后产能涉及企业数占全省三分之一，提前完成“十二五”落后产能淘汰任务。正泰集团、华峰集团入选全省首批“三名”试点企业，新增产值超10亿元企业6家、超亿元企业46家。提质推进“三转一市”。完成“个转企”1.8万家、“小升规”958家、“企转股”230家，均居全省第一；新增上市企业2家，在新三板和区域性股权交易平台挂牌企业110家。直接融资增长43.6%，中长期贷款增长30.6%，融资结构日趋合理。

　　（四）城乡建设有力推进。都市基础设施网络加快构建。甬台温高速复线、绕城高速西南线和北线二期加快推进，龙丽温（泰）高速瑞安至文成段动工建设，诸永高速延伸线（瓯越大桥）建成通车；市域铁路S1线建设全线铺开，金温铁路扩能改造工程基本具备铺轨条件；乐清湾港区一期建成开港；龙湾国际机场新飞行区投用，T2航站楼加快实施。乐清湾港区铁路支线、状元岙港区二期、东部综合交通枢纽、鳌江四桥等项目开工建设。瓯飞一期、瓯江口二期等项目加快推进，瓯飞起步区堵口提前合龙。平阳顺溪水利枢纽下闸蓄水，永嘉县城排涝应急工程投用。获批信息惠民国家试点城市，4G网络实现乡镇全覆盖，市区主要公共场所开通免费WiFi。深化电网建设攻坚，500千伏市域环网基本形成；东海丽水36-1气田建成投产。争取重大基础设施用地指标2.6万亩；争取建设用海指标1.3万亩，占全省近一半。都市核心城区加快提升建设。“3+1”亮点区块建设扎实推进，滨江商务区18幢大楼主体结顶或投用，中央绿轴公园启动建设，三垟湿地公园加快实施，鹿城双屿综合整治全面推开；龙湾中心区、瓯海中心区、瓯江口新区、经开区滨海新城、永嘉三江口片区等功能区块提速推进；鹿城“黎明·92”等旧厂区时尚化改造项目初展形象，市区六大主入城口环境综合整治和道路彩化提升初见成效。深入推进城市治堵，打通市区惠民南路等卡口11个、断头路10条，开工首条BRT专线，新增公交专用道30公里、自行车专用道74公里、停车泊位1.8万个，公交分担率提高3个百分点。城乡协调发展得到加强。完成温州都市区规划纲要编制，城市总体规划修编上报国务院审批。组团城市建设整体推进，各县（市）新城区靓丽形象逐步呈现，瑞安入选全省首批美丽县城试点县。推出文成、泰顺“工业飞地”机制，两县列入国家主体功能区建设试点。龙港镇列入国家新型城镇化综合试点，大峃镇、罗阳镇列入省级小城市培育试点。建成美丽乡村示范村133个、精品线15条。创成新农村电气化市。城乡统筹发展水平提升幅度居全省第一。

　　（五）环境面貌大有改观。狠抓“六城联创”。创成国家森林城市、国家卫生城市、全国文明城市，国家园林城市、国家环保模范城市、国家历史文化名城创建取得积极进展。狠抓“五水共治”。完成治水投资248亿元，居全省第一。基本消灭垃圾河，整治黑臭河398条530公里；基本建成镇级污水处理厂23座，占全省三分之一；完成生活污水全面治理村建设1101个，占全省六分之一；市区101个片区截污纳管工程有序推进，建成一、二级污水管网396公里。全市平原河网主要污染物平均浓度明显下降，珊溪库区黄坦坑入库口水质根本性好转。获批全国水生态文明试点城市。瓯海、永嘉荣获全省“五水共治”优秀县（市、区）“大禹鼎”。狠抓“三改一拆”“四边三化”。开展“无违建”创建，依法推进涉宗违建处置，拆除各类违法建筑1341万平方米，实施“三旧”改造5659万平方米。建成滨水公园178座；圆满完成绿道网三年建设任务，总长度达3668公里。加快修复振兴“浙南鱼仓”，“一打三整治”完成率居全省第二。狠抓汽车尾气、扬尘烟尘等治理，淘汰黄标车和老旧车辆4.9万辆，市区大气环境优良天数达300天，PM2.5浓度下降幅度、生态环境质量公众满意度提升幅度均居全省第一。

　　（六）民生福祉持续提升。认真办好民生实事。千方百计兑现政府承诺，年度十个方面、20项实事和“安置提速”三年任务基本完成，累计完成营业房安置3554间、住宅交钥匙14099套，办理“两证”18906个。全力实施大病保险制度，惠及600多万参保人员；妥善解决15万名被征地农民生活保障和5万名改制企业人员门诊统筹问题；城乡居民基本养老保险月均养老金提高23%以上，企业退休人员基本养老金平均提高10%。繁荣发展社会事业。6个县（市、区）通过国家义务教育基本均衡评估认定，创成省级等级幼儿园164所、省级义务教育标准化学校169所。温州肯恩大学正式建校，浙江安防学院（筹）一期投用。推进优质医疗资源“双下沉、两提升”，分级诊疗试点稳步推开。9个县（市、区）创成省级卫生强县。市急救中心、市六医一期投用。“单独两孩”政策顺利实施。成功承办第三届中国越剧节、中华龙舟大赛和省第五届少数民族传统体育运动会，建成农村文化礼堂290个、社区文化活动中心306家，万人拥有文化设施面积居全省第二。全力争取平安大市“六连创”。开展十大重点领域安全生产专项整治，安全生产三项主要指标同比持续下降。深化社会治安“六防工程”，刑事、治安、“两抢”案件分别下降6.2%、10.3%和48.4%。一批久而未决的历史遗留问题和信访积案得到化解。全力做好文成、泰顺地震救灾工作。支持国防和军队建设。工会、共青团、妇幼、老龄、慈善、残疾人等事业全面发展，民族、宗教、外事、侨务、扶贫等工作取得新成绩。

　　过去一年，全市政府系统扎实开展党的群众路线教育实践活动，切实加强政府自身建设，群众对政府服务满意度较上年提高4.7个百分点。深化依法行政。积极争取地方立法权。完善法治政府建设考评体系。主动接受人大依法监督和政协民主监督，认真执行人大各项决议决定，办理人大代表建议513件、政协提案556件，满意率98%以上。提升行政效能。深入开展“万人评议机关中层和基层站所”活动，公共服务窗口推行办公无休日制度。实施“电视问政”“网络问政”，上线“温州发布”“政策速递”，推行“微信工作法”。严格正风肃纪。坚决落实中央“八项规定”、省委“六个严禁”，市县两级政府“三公”经费支出下降32.7%，办公用房清理整改到位。加大监察问责力度，集中整治了一批“四风”问题，严肃查办了一批违法违纪案件。

　　各位代表，过去一年的成绩来之不易，这是全市上下齐心协力、克难攻坚的结果。在此，我代表市人民政府，向在各个领域辛勤工作、作出贡献的全市人民，向给予政府工作大力支持的人大代表、政协委员，向所有参与、关心、支持温州建设发展的社会各界人士和海内外朋友们，致以崇高的敬意和衷心的感谢！

　　我们也清醒地看到，我市经济社会发展还存在不少问题，政府工作还有许多不足。一是经济发展尚未走出困境，“两链三险”依然错综复杂，不少企业生产经营困难，经济下行压力较大，虽然与全省发展差距缩小，但是去年地区生产总值、进出口总额两项指标仍没有达到预期目标。二是产业转型升级不快，“一产不精、二产不强、三产不兴”问题较为突出，科技、人才支撑不力仍然是我市发展的致命弱点。三是城乡区域发展不平衡，中心城市能级不高，“半城市化”现象严重，生态环境整治和环保基础设施建设任务艰巨。四是社会事业发展水平有待进一步提高，一些与群众切身利益相关的问题没有得到及时有效解决。五是政府自身建设仍需加强，一些体制机制没有理顺到位，一些政府工作人员改革意识、法治意识、责任意识、作为意识不强，一些部门和地方“四风”问题依然存在，一些领域消极腐败现象时有发生。对这些问题，我们一定高度重视，采取有力措施逐步加以解决。

二、2015年总体思路和主要目标任务

　　2015年是完成“十二五”规划、谋划“十三五”发展的承上启下之年，是全面深化改革、加快赶超发展的攻坚突破之年。当前，全国经济发展进入新常态，呈现速度变化、结构优化、动力转换的特点。温州作为市场经济先发早发地区，较早遇到转型的阵痛，深受“三期叠加”的影响。必须加快重构新动力。坚持质量效益引领发展，突出制度供给，强化创新驱动，向改革开放要红利、向科技人才要活力、向结构调整要助力，打造动力强劲的新引擎。必须着力提升新优势。顺应时代潮流，对接国家战略，加快培育提升新的区位条件优势、体制机制优势、产业竞争优势和创业创新优势，努力在激烈的区域竞争中赢得主动。必须奋力推进新发展。组织实施生态化、信息化、时尚化、都市化、国际化“五化战略”，全面推进产业、企业、市场、城市“四大转型”，大力发展新产业、新技术、新业态和新商业模式，加快建设山水智城、时尚之都、美丽温州。

　　按照市委十一届八次全会部署，今年政府工作的总体要求是：全面贯彻党的十八大和十八届三中、四中全会精神，坚持以习近平总书记系列重要讲话精神为指导，坚持以“八八战略”为总纲，坚持“稳中求进、克难奋进”工作基调，按照省委、省政府的重大决策部署和“干好一三五、实现四翻番”的要求，实施“五化战略”，深化“十大举措”，全面深化改革开放，全面加快转型升级，全面推进法治建设，全面保障改善民生，全面提升政府效能，努力开创新常态下温州赶超发展新局面，不断推进“温州模式”创新发展。

　　建议今年全市经济社会发展的主要预期目标为：地区生产总值增长7%以上；一般公共预算收入增长7%以上；限上固定资产投资增长12%以上；社会消费品零售总额增长11%以上；外贸出口增长5%左右；城镇和农村常住居民人均可支配收入分别增长8%以上、9%以上；节能减排完成省下达任务。

　　重点抓好八个方面工作：

　　（一）全面深化改革开放，不断提升发展活力。

　　狠抓政府自身改革。建立健全“四张清单”动态调整机制，确保真放权、放实权；再削减一批行政权力，再清理和下放一批市级审批事项，实现非行政许可审批事项清零，推广负面清单外企业投资项目不再审批，在永嘉和高新区开展市县同权扁平化改革试点。推进环评审批制度改革。按照“养事不养人”和“费随事转”的思路，深化政府职能向社会组织转移“第五张清单”改革，加大政府向社会力量购买服务力度。注重放管结合，加强事中事后监管。拓展提升政务服务网功能，推进部门审批系统并网。实施新一轮政府机构改革，严格控编控人控机构，科学设置部门内设机构和流程；抓好事业单位分类改革。编制三年滚动财政规划，逐步建立跨年度预算平衡机制。扩大部门预决算公开范围。探索财政专项资金透明化、竞争性分配改革。实施新一轮市区财政体制和城建管养体制。做好政府性债务融资方式转变和平稳过渡工作。

　　推进全方位改革。实行“一地一试点、一行一创新”，组织实施重点改革项目攻坚。深化经济体制改革。加大要素配置市场化改革力度，抓好乐清、瑞安省级扩面试点，创造条件向全市推开。推进商事制度改革，支持瓯海开展市场准入“多证合一”试点。建立机场、港口、铁路、公路投融资良性机制，推广政府与社会资本合作模式，探索设立投资引导基金，推出社会投资指导目录。优化国企法人治理结构和运行机制，推进国有资产证券化、国有企业股份制改造，发展混合所有制经济。深化社会体制改革。加大民办社会事业改革力度，引导更多社会资本进入教育、医疗、养老等领域，切实把“全国试点”办成“全国示范”。推动行业协会（商会）、中介机构规范化建设，提升社会组织承接政府职能、参与社会治理的能力。加强专业化社工队伍建设。深化金融综合改革。制定新一轮金融综合改革实施意见，加大金融服务实体经济力度。做大做强地方金融板块，推动设立地方性商业银行、证券公司、保险公司，组建地方金融资产管理公司。推进地方金融资产交易平台建设。创建企业融资地方增信体系，深入实施普惠金融工程。规范和发展民间融资，有序发展互联网金融。深化农村综合改革。加大农村资产确权赋权活权改革力度，积极推进“三权到人（户）、权跟人（户）走”，加强村股份经济合作社规范化建设，提升土地经营权流转水平，农户宅基地发证率达85%以上。抓好龙港镇国家新型城镇化综合试点。支持平阳深化户籍制度改革。

　　进一步提升对外开放水平。积极参与“一带一路”、长江经济带等国家战略，加快谋划建设温州联系浙中、通往中西部地区的铁路通道，推进浙闽省际交通建设，提升空港、海港国际化水平，着力打造“一带一路”节点城市和长江经济带通海门户。主动承接上海自贸区可复制改革试点经验。支持温州企业加快“走出去”，在“一带一路”沿线国家布局产业基地、商业网络。推动乌兹别克斯坦鹏盛工业园创建国家级经贸合作区。全方位展开对韩开放合作，高起点开发建设“东方时尚岛”、温州韩国产业园，狠抓医乐园、韩国时尚新天地等重大项目落地。深化浙台（苍南）经贸合作区建设，加快创建海峡两岸（温州）民营经济创新发展示范区。积极参与长江经济带区域通关一体化改革，加快电子口岸建设，确保保税物流中心（B型）通过验收，争取创成国家跨境电子商务试点城市，加快乐清湾港区对外开放，积极申报综合保税区。做好对口支援工作。

　　（二）突出创新驱动发展，增强城市核心竞争力。

　　积极搭建公共创新平台。提升国家级高新区、大学科技园发展水平，加快规划建设浙南科技城，鼓励有条件的地方争创省级高新园区。大力支持在温高校发展科研事业，发挥地方科技资源作用，推进科研院所科研仪器设备向社会开放。深化产学研合作，提升发展激光与光电、泵阀等产业研究院，办好华中研究院、联合研究院、北航温州研究院、中津先进科技研究院等创新平台，确保中科院温州生材所落地建设。

　　发挥企业创新主体作用。引导企业加大科技投入，新增高新技术企业100家、科技型中小企业500家以上，新建省级企业研发中心20家，培育省级技术中心、产业技术联盟5家。加快发展网上技术市场，促进科技成果转化。深化国家科技金融结合试点，探索设立种子基金、创业基金等，助力创新型中小微企业加速发展。研究与试验发展经费支出占生产总值比重达2%左右。

　　加大人才培养引进力度。把人才作为推动创新发展的首要因素，成立市招才局，出台“人才新政”，狠抓高层次人才引进和高技能人才培养，广泛动员温籍英才助力家乡发展。新入选“国千”“省千”15人，培养高技能人才2万人。优化创业创新环境，推动形成大众创业、万众创新的生动局面。

　　提升质量强市建设水平。以国家质量强市示范城市建设为抓手，制定实施品牌培育、知识产权保护、标准化建设行动计划，抓好国家级质检中心建设，严明质量执法，推动“温州制造”向“温州智造”提升。

　　（三）推动经济协调发展，开创稳定增长新局面。

　　扩大有效投资。持续推进“赶超发展三年超万亿投资计划”，完成限上固定资产投资3420亿元，民间投资比重达62%。坚持全市招商引资“一盘棋”，持续推进“温商回归三年超三千亿工程”，紧盯“温商领头雁”，发挥异地温州商会和招商专员作用，开展点对点精准招商，促进贸易回归、总部回归、高端项目回归，争取引进一批20亿元以上重大产业项目，确保到位资金1000亿元。抓好转而未供、供而未用土地清理，实施城镇低效用地再开发2.1万亩、完成9000亩；争取建设用海指标1万亩。

　　扩大社会消费。完善促进消费政策措施，积极培育信息（网络）消费、时尚消费、健康消费、文化消费、老龄消费等消费热点。推动城市商圈转型发展，加快环五马时尚商业区改造升级。实施月光经济发展三年行动计划，打造一批品质夜市街区和夜游线路。优化商贸综合体布局，实现差异化发展。加强社区商业网点、快递投送网点等便民商业设施建设。加快专业市场“迁改并转”。鼓励传统商业发展O2O模式，促进线上线下消费融合。推动瓯菜振兴，促进餐饮业发展。实施电子商务进万村工程，健全农村现代流通网络，激活农村消费市场。

　　扩大外贸进出口。坚持出口、进口、转口贸易发展并重，制定促进外贸综合服务企业发展政策措施，推进出口商品质量安全示范区建设。深化与中信保合作，建立成长型出口企业政府联保平台，鼓励企业抱团参展，拓展国际市场。优化通关服务、退税服务，联动推进跨境贸易人民币结算。用好侨务资源，促进侨贸转型升级。依托温州人网络培育服装、箱包、葡萄酒等进口名品市场，打造国际时尚消费品进口贸易高地。

　　狠抓风险防范化解。统筹运用应急转贷、重组清算、资产盘活等手段，继续发挥市县两级处置办作用，加强风险企业分类帮扶处置，着力化解和压缩银行不良贷款；积极引导流动性注入，建立小微企业信保基金，优化小额贷款保证保险，做大做强国有担保公司和再担保中心，扩大抵（质）押物范围，新增金融融资规模600亿元以上，信贷增长力争达到全省平均水平，努力实现从“风险先发”到“率先突围”转变。坚持供地把控、政策调控、功能配套、市场监管、舆论引导多措并举，稳定住房消费预期，促进房地产市场健康发展。

　　（四）加快振兴实体经济，着力构建现代产业集群。

　　提升特色产业发展水平。大力发展时尚产业，坚持品牌、品质、品位“三品”齐抓，统筹抓好时尚街区打造、时尚项目落地、时尚品牌培育、时尚产品研发，推动轻工产业向时尚产业转型。大力发展信息经济，积极培育云计算、大数据、物联网，加快发展电子商务、信息产品制造、软件等产业。大力实施“五一○产业”发展规划、行动计划，加快传统支柱产业改造升级，推动电气产业向智能装备产业发展、鞋服产业争当时尚产业排头兵、汽摩配产业与整车机车生产企业紧密对接、泵阀产业向系统流程装备产业转型。培育发展网络经济、旅游休闲、现代物流、激光与光电、临港石化、轨道交通、通用航空、新材料、文化创意、生命健康十大新兴产业，加快形成新的产业优势。大力发展绿色建筑，加快建筑业转型升级。做精做专电镀、模具、铸造等产业，把基础做实、配套做全，打造“引不走”的产业集群。

　　持续深入开展工业创强。突出工业主导地位，狠抓工业强县建设，发挥乐清省级工业强县示范带动作用，支持瑞安、龙湾（包括经开区）创建省级工业强县，鼓励瓯海、永嘉向规上工业产值超500亿元冲刺。狠抓工业强镇建设，建成规上工业产值超100亿元强镇12个。狠抓工业强企建设，加大龙头企业、成长型企业培育力度，开展“亿元工业企业冲千家”专项行动，新增产值超亿元企业100家。深入实施“三转一市”，加强现代企业制度建设，健全“个转企”机制，完成“小升规”500家、“企转股”100家，推动80家企业上市和在新三板、区域性股权交易平台挂牌。深入实施“四换三名”，推进信息化与工业化深度融合，抓好500项“机器换人”项目，培育信息工程公司20家以上，完成技改投资620亿元；促进电商普及应用，争取网络零售额突破1000亿元；鼓励企业开展节地改造和二次开发；推进落后产能淘汰，全面完成六大重污染、高耗能行业整治。

　　加快发展现代服务业。优先发展生产性服务业，实施现代物流业发展三年行动计划，着力提升科技研发、现代金融、商务服务、中介服务等发展水平，积极培育总部经济、楼宇经济，引进高端产业综合体，加快构建产业生态圈。大力发展旅游经济，深化A级旅游景区创建，推进雁荡山-楠溪江、环飞云湖旅游一体化，加快省级旅游度假区开发，做深做好海岛海洋旅游文章；狠抓重大旅游项目建设，提升景区信息化水平，推进自助旅游营地驿站创建，努力打造国内外知名优秀旅游目的地城市。加快发展会展经济，积极引进国际知名会展公司，扩大时尚产业博览会影响力。多模式发展生活性服务业。鼓励有条件的地方争创省级服务业强县。

　　积极发展现代农业。强化耕地保护，稳定粮食播种面积，着力提升储粮能力，保障“菜篮子”供应。建成高标准基本农田15万亩、粮食生产功能区10万亩、现代农业园区30个。做大做强现代农业经营主体，新增家庭农场500家。加强农合联组织建设，培育“三位一体”示范性农民合作组织30家、区域性农业综合服务中心20个。深化农村金融体制改革，规范提升农村资金互助组织50家。推进农业“种、养、加、销”一体化发展，打造产值超10亿元农业全产业链集群5个。实施“科技兴农”，加快发展高效生态循环农业。推进都市农业、精品农业发展，开展“美丽田园”创建，建设一批时尚农业产业基地。做好山地文章，发展现代林业。积极培育渔港经济区，促进渔业经济健康可持续发展。加强动植物疫病防治。扩大政策性农业保险覆盖范围。

　　加强产业功能平台建设。理顺瓯江口产业集聚区、浙南沿海先进装备产业集聚区、温州经济技术开发区管理体制，加大开发建设力度，狠抓大项目引进和落地，早日成为赶超发展的新增长极。深化开发区（工业园区）整合提升，支持乐清经济开发区申报国家级开发区，推动有条件的工业园区整合为省级开发区。加快建设、投产一批小微企业园。大力发展海洋经济，提升四大海洋特色产业基地开发水平，支持洞头省级海岛综合开发与保护试验区加快建设。

　　（五）强化区域城乡联动，提升温州都市区建设水平。

　　推进组团城市开发建设。实施温州都市区规划纲要，完成城市总体规划、土地利用总体规划报批，推进“多规合一”。按照“一主两副三极多点”空间布局，做精做强鹿城、龙湾、瓯海三大城区，推进市区与瑞安、乐清、永嘉区域融合发展，加快洞头改县设区步伐，着力构建中心城区新格局。推进鳌江两岸一体化发展，支持平阳苍南加快建设都市区副中心。支持文成、泰顺深化国家主体功能区建设，努力打造都市区生态高地。推进“靓丽县城”建设，抓好省级小城市培育试点，推动农业转移人口向城镇集聚。支持瓯海、永嘉、泰顺、苍南等地开展省级特色小镇建设，着力培育一批市级特色小镇、时尚小镇。

　　健全都市基础设施网络。围绕建设综合交通网，加快实施甬台温高速复线、绕城高速西南线和北线二期、龙丽温（泰）高速瑞安至文成段，确保龙丽温（泰）高速泰顺支线获国家批准；加快实施市域铁路S1线、乐清湾港区铁路支线，开工建设市域铁路S2线，金温铁路扩能改造工程建成通车；加快实施龙湾国际机场T2航站楼、通用航空基地；统筹抓好乐清湾、状元岙、大小门岛三大核心港区建设。大力推进东部综合交通枢纽、瓯江北口大桥、七都大桥北汊桥、鳌江三座桥和一批国省道项目，330国道鹿城段、大门大桥建成通车。积极谋划温义合高铁（杭温客专），深化城市轨道线、温衢丽铁路、温武铁路等项目前期。围绕建设能源保障网，投产3个500千伏、10个220千伏输变电工程，大力推进浙能乐清电厂三期、中石化LNG及核电、风电、水电、抽水蓄能等项目。围绕建设现代水利网，加快瓯江、飞云江、鳌江流域综合治理和独流入海项目建设，抓好市区百年一遇防洪堤、温瑞平原排涝、永嘉南岸水库等水利项目；加快实施瓯飞一期、瓯江口二期等围垦工程，提高围区开发建设水平。

　　提升城市功能品位。加强城市设计，严把重点区块和地标建筑方案审查关。聚焦“3+1”亮点区块建设，中央绿轴公园建成开园，滨江商务区沿江片、三垟湿地公园初显靓丽形象，鹿城双屿综合整治实现更大突破。理顺江心屿管理体制，加大保护开发力度，焕发名屿昔日风采。坚持“三改一拆”方向不变、力度不减，完善城中村改造和“退二进三”政策，探索市场化安置等办法；深化“无违建”创建，加快拆后土地利用。完成市区主入城口环境综合整治。加强大罗山保护管理开发。加快“智慧温州”建设，推进光纤到户、WiFi热点覆盖和通信铁塔集约化；组建大数据管理中心，市县两级共建电子政务云；抓好信息惠民、智慧旅游国家试点，加强信息技术在教育、医疗、交通、城市管理等领域的推广应用，建成地下管网信息管理系统。深入实施交通拥堵治理工程，开通公交线路实时查询系统，建成BRT一号线。完善城市慢行系统，统筹推进自行车道、步行道、绿道建设。加快城市快速路、主干道路建设，促进城市组团间融合。继续实施“打卡口、接断路”工程，完善城市路网，方便市民出行。加快核心区停车设施建设，开展老旧住宅区交通微循环改造，努力缓解停车难。启动城市垃圾分类处理试点。加大工业垃圾、建筑垃圾处理力度，加快建设西向生态填埋场，建成综合材料生态处置中心、永强垃圾焚烧厂二期，开工建设餐厨垃圾处理厂。

　　支持农村和山区加快发展。深化美丽乡村建设，建成精品村100个、精品线10条。启动实施农村公路发展三年行动计划，提高公路等级和行政村通班车率。深入实施农村饮水安全工程，着力解决山区城镇集中供水问题，提高农村供水保证率和饮水安全覆盖面。深化“山海协作”，加快推进文成、泰顺“工业飞地”建设，推动偏远山区、海岛地区、革命老区加快发展。认真落实全省民族地区发展六大计划。依法加强饮用水水源地保护，完善生态补偿机制，支持库区群众转产转业，努力实现保护一缸清水与致富一方百姓的双赢。大力支持文成、泰顺抗震安居工程建设。加大扶贫攻坚力度，深入实施“低收入农户收入倍增计划”；启动25个村整村规模搬迁，完成7000人异地搬迁集聚。

　　（六）狠抓生态环境整治，加快建设美丽温州。

　　全面推进水环境综合整治。保持“五水共治”攻坚态势，认真落实全省消灭黑臭河和劣V类水三年行动计划，城区基本消灭黑臭河。实施温瑞主塘河“一环三线”水岸同治三年行动计划，加快打造美丽水乡核心区。开工建设市区中心片污水处理厂迁建工程，加快建设西片污水处理厂，建成南片一期、东片二期污水处理厂；新增生活污水全面治理村和扩面改造村1596个；同步推进截污纳管，新增一、二级污水管网290公里，着力解决“最后一米”纳管问题，市区污水处理率达92%，城镇污水基本实现“应收尽收、应处尽处”。加强海洋生态保护，联动推进乐清湾、瓯江口等近岸海域污染防治。深入开展“一打三整治”专项行动，促进“浙南鱼仓”修复振兴。

　　全面推进大气污染防治。以雾霾治理为重点，深入实施“六大行动”，认真落实全省燃煤电厂清洁排放技术改造三年行动计划，加快燃煤锅炉淘汰，各县（市）建成区完成大气重污染企业关停搬迁。加强机动车尾气污染防治，基本淘汰黄标车。统筹抓好餐饮油烟、垃圾焚烧、建筑工地与道路扬尘等治理，扩大清洁能源利用，进一步改善空气质量。

　　全面推进土壤污染治理。认真落实全省农业“两区”土壤污染防治三年行动计划。强化土壤污染源排查和监控，开展污染场地治理修复示范工程建设。推进危废和污泥精细化管理，实现垃圾焚烧飞灰无害化处置。

　　全面完成“六城联创”目标。力争创成国家园林城市、国家环保模范城市、国家历史文化名城，巩固提升国家森林城市、国家卫生城市、全国文明城市创建成果。支持文成创建国家级生态县。深入推进“两无三化”，提升绿化层次，加强后期管养，新增绿化造林面积6万亩、城市绿地330公顷。完成滨水公园三年建设任务。认真贯彻实施新环保法，加大环保执法力度，对一切环境违法行为“零容忍”。

　　（七）大力发展社会事业，提高群众幸福指数。

　　围绕群众所思所盼，健全为民办实事工作机制，认真办好社会关爱、便民服务等十个方面民生实事，以实际成效造福于民。

　　加快发展教育事业。实施学前教育第二轮三年行动计划，等级幼儿园覆盖率达85%。启动薄弱学校改造提升、推进优质教育资源均衡发展三年行动计划，创建省义务教育标准化学校100所。深化普通高中特色示范创建。大力发展职业教育，加快建设滨海职教中心，支持有条件的地方创办高职院校。支持温州医科大学列入省重点高校建设计划，办好温州大学，加快温州肯恩大学、浙江安防学院（筹）、温州科技职业学院校园建设，推进温医仁济学院、浙江工贸学院、浙江东方学院等院校迁扩建。加强教师队伍建设，不断提高教育教学质量。

　　加快发展卫计事业。深化医药卫生体制改革，建立医疗资源下沉长效机制，构建基层首诊、双向转诊、分级诊疗的就医制度。加快附二医瑶溪新院、市人民医院娄桥新院、市七医、市中心血站、温州护士学校等项目建设，建成市中医院新院一期、市妇幼保健院综合大楼和一批县（市）医院。开展群众满意乡镇卫生院创建。建成省级卫生强市。加大全科医师培养引进力度。加强传染病和慢性病防控。完善医疗纠纷调处机制，构建和谐医患关系。推进卫计资源整合，深化出生人口性别比综合治理，提升计生优质服务水平。

　　加快发展文体事业。注重文化滋养，大力弘扬社会主义核心价值观，深化“争做最美温州人”等主题活动。做深“瓯”系列文章，推动优秀传统文化传承与创新。保护好一批具有浓厚历史积淀的古村落、古民居、古建筑，打造一批特色侨乡、侨村，让在外温州人记得住乡愁。加强名人故居保护修缮。继续推进雁楠、廊桥、矾矿申遗。加大文体惠民力度，建成市文化艺术大楼，推动一批县级文化中心、专题博物馆投用，抓好农村文化礼堂和城市文化公园建设。办好第五届艺术节、第三届市民文化节等活动。广泛开展全民健身运动，扩大学校体育场地设施向社会开放。依托秀美山水资源，积极谋划开展山地户外运动、马拉松赛事等时尚文体活动。建成奥体中心主体育场一期、市水上运动训练基地一期。加强文艺精品创作。支持培育具有温州特色的影视产业，鼓励发展工艺美术、3D设计、微电影等文创产业。支持国家广告产业试点园区建设。

　　提升社会保障水平。实施全民参保登记，推进社保从制度全覆盖向人员全覆盖提升。抓好机关事业单位工作人员养老保险制度改革。探索“医养融合”养老模式，加快多功能护理型养老机构建设，新增床位5000张；深化公办养老机构改革，新建城乡居家养老服务照料中心600个。推行“市场提供房源、政府补贴租金”保障模式，新增公共租赁房货币化补贴保障家庭2000户。完善社会救助体系，深入开展全国“救急难”试点工作。发展红十字、慈善等公益事业，切实保障妇女、儿童和残疾人合法权益。加大“青山白化”治理力度，深化绿色生态殡葬改革。

　　（八）推进社会治理创新，保持社会和谐稳定。

　　提升社会治理水平。完善乡镇（街道）与功能区体制。推进社区规范化建设，完善网格化管理体系，提高基层社会自我管理服务能力。发挥市民公约、村规民约在城乡治理中的积极作用。落实“7431”网络管理新模式，净化网络空间。依法加强宗教事务管理，深入开展“同心同行·共建和谐”活动。发挥老干部作用。支持工会、共青团、妇联、残联等人民团体广泛参与社会管理和公共服务，做好外事侨务、统计、档案、地方志等工作。

　　深化平安大市创建。完善立体化社会治安防控体系，依法防范和惩治违法犯罪活动。加大打黑除恶力度，净化社会环境。深入开展十大重点领域安全生产专项整治，坚决遏制重特大事故发生。强化食品药品和农产品质量安全监管，保障人民群众“舌尖上的安全”。推行“阳光信访”。深化人民调解、行政调解、司法调解工作。健全社区矫正管控机制。加强法律援助、司法救助，完善公共法律服务体系。妥善处置劳资纠纷。强化应急管理，引入航空应急救援，提高突发事件应对能力。加强国家安全工作。

　　提升信用温州形象。坚持以政务诚信示范引领全社会诚信建设，加快构建政务诚信、商务诚信、社会诚信和司法公信“四位一体”社会信用体系。发挥人行征信中心温州分中心作用，建好信用信息共享平台，发展第三方征信机构，建立统一的失信“黑名单”制度。加强诚信教育，强化规则意识，倡导契约精神，严厉打击逃废债、无理“房闹”、发布虚假广告等行为，着力营造诚信光荣、失信可耻的浓厚氛围。

　　支持驻温人民解放军和武警部队建设，推进国防后备力量和人民防空建设，实现全国双拥模范城市“四连冠”。

　　三、突出法治政府建设主线，加强政府自身建设

　　认真贯彻落实市委《关于全面深化法治温州建设的意见》，加快建设职能科学、权责法定、执法严明、公开公正、廉洁高效、守法诚信的法治政府，推动政府治理体系和治理能力现代化。

　　坚持依法行政。出台《深入推进依法行政加快建设法治政府的实施意见》。坚持法定职责必须为、法无授权不可为，依法全面履行政府职能。自觉接受人大依法监督、政协民主监督，积极听取民主党派、人民团体和各界人士的意见建议。加大政务公开力度，主动接受社会监督、舆论监督。加强监察、审计工作。深化行政执法体制改革，加快推进综合执法。建立执法全过程记录制度，推广行政处罚裁量基准制度，促进严格规范公正文明执法。探索行政复议体制改革，坚决纠正违法或不当行政行为，切实降低行政诉讼败诉率。

　　坚持科学施政。完善公务员学习培训制度，强化理论武装和业务学习，提高依法办事、为民服务的意识和能力。完善市政府常务会议集体学法制度，加强行政机关负责人法律培训。坚持问题导向，注重调查研究，健全重大行政决策制度，把公众参与、专家论证、风险评估、合法性审查、集体讨论决定作为重大决策必经程序，提高科学民主决策水平。坚持问政于民、问需于民、问计于民，加强智库建设，全面推行法律顾问制度。继续争取地方立法权，加快建设与之相适应的政府法制机构和专业队伍。

　　坚持效能促政。强化政府抓落实的工作属性，组织开展扩大有效投资、“温商回归”落地、小微企业园建设和工业技改、企业金融风险化解、市区“3+1”亮点区块建设、“水岸同治”重点片区建设、“六城联创”、民生实事工程等经济社会发展“八大攻坚”行动。强化重要决策部署和重大事项推进的督查落实，严格行政问责，确保政令畅通，切实提高政府执行力和公信力。建立健全区域联动发展机制，加强对县际重大事务的协调和处置。深化“反庸治懒”专项行动，着力解决不担责、不作为等问题。改进考核考绩工作，优化考核指标体系，进一步形成良好的考核评价导向与干事创业氛围。

　　坚持廉洁从政。巩固和拓展党的群众路线教育实践活动成果，扎实开展“三严三实”专题教育，持之以恒抓好“四风”问题整改，推进作风建设制度化、规范化、常态化。全面开展机关事业单位“吃空饷”问题集中整治。深化公务用车制度改革，严控“三公”经费支出。加强反腐倡廉制度建设，严格落实“一岗双责”，严肃查处违法违纪案件，着力营造干部清正、政府清廉、政治清明的良好风尚。

　　各位代表！认真研究制定“十三五”经济社会发展规划，是今年的一项重要工作。我们将以改革思路、法治思维、战略眼光，认真谋划好重大工程、重大项目、重大政策，为实现更高水平、更好质量、更可持续发展打下坚实基础。

　　各位代表！新常态，新愿景，新使命。让我们紧密团结在以习近平同志为总书记的党中央周围，在省委、省政府和市委的坚强领导下，团结和依靠全市人民，在深化改革中抢抓机遇、开拓进取，在攻坚克难中爬坡奋进、精准发力，为温州“赶超发展、再创辉煌”而努力奋斗！
